# *NRAMP1* and *VDR* Gene Polymorphisms in Susceptibility to Tuberculosis in Venezuelan Population

**DOI:** 10.1155/2015/860628

**Published:** 2015-10-22

**Authors:** Mercedes Fernández-Mestre, Ángel Villasmil, Howard Takiff, Zhenia Fuentes Alcalá

**Affiliations:** ^1^Laboratorio de Fisiopatología, Centro de Medicina Experimental “Miguel Layrisse”, Instituto Venezolano de Investigaciones Científicas, Kilómetro 11 Carretera Panamericana, Apartado 21827, Caracas 1020A, Distrito Capital, Venezuela; ^2^Laboratorio de Genética Molecular, Centro de Microbiología y Biología Celular, Instituto Venezolano de Investigaciones Científicas, Kilómetro 11 Carretera Panamericana, Apartado 21827, Caracas 1020A, Distrito Capital, Venezuela; ^3^Unidad de Tórax, Hospital Dr. José Ignacio Baldó “El Algodonal”, Avenida Intercomunal de Antímano, La Yaguara, Apartado 1000, Caracas 1020A, Distrito Capital, Venezuela

## Abstract

Natural resistance-associated macrophage protein (Nramp1) and the vitamin D receptor (VDR) are central components of the innate and adaptive immunity against *Mycobacterium tuberculosis*, and associations between susceptibility to tuberculosis and polymorphisms in the genes *NRAMP* and *VDR* have been sought in geographically diverse populations. We investigated associations of *NRAMP1* and *VDR* gene polymorphisms with susceptibility to TB in the Venezuelan population. The results suggest the absence of any association between *VDR* variants *FokI, ApaI*, and *TaqI* and susceptibility to tuberculosis. In contrast, the *NRAMP1 3′UTR* variants were associated with susceptibility to *M. tuberculosis* infection, as seen in the comparisons between TST+ and TST− controls, and also with progression to TB disease, as shown in the comparisons between TB patients and TST+ controls. This study confirms the previously described association of the *NRAMP1 3′UTR* polymorphism with *M. tuberculosis* infection and disease progression.

## 1. Introduction

Tuberculosis (TB) is the second cause of death worldwide among infectious diseases. In the World Health Organization (WHO) Global Tuberculosis Report for 2014 there were 9.0 million new TB cases in 2013 and 1.5 million TB deaths (1.1 million among HIV negative and 0.4 million among HIV positive), of which 218,875 new cases and relapses occurred in the Americas. In Venezuela, according to the WHO, the incidence of new and relapse TB cases is between 20 and 49 per 100,000 inhabitants [1]. The number of subjects infected with* Mycobacterium tuberculosis* (Mtb) is much higher, but the great majority of those infected are able to keep the pathogen under control and never develop the disease. Multiple evidence suggests that there is a genetic component involved in determining resistance or susceptibility to TB patients, who are infected but do not develop the disease and who developed the disease, but it is difficult to conduct genetic studies on susceptibility to infectious diseases because of the multifactorial influences of the host, the pathogen, and environmental variables that differ for each disease and even each individual studied. The association of host genetic factors with susceptibility or resistance to TB has been studied extensively using various methods including case-control studies, candidate gene approaches, and family-based and genome-wide linkage analyses that have revealed several candidate genes involved in susceptibility (reviewed in [2]). These studies have been performed in different ethnic groups, with large discrepancies between groups regarding the effect of the different candidate genes. Two of the genes that have shown the most robust associations are* NRAMP1* and* VDR*.

In this study we investigated associations of* NRAMP1* and* VDR* gene polymorphisms with susceptibility to TB in the Venezuelan population.

## 2. Material and Methods

### 2.1. Subjects

The study included one hundred and ninety-five (195) unrelated and ethnically mixed Venezuelan individuals from the northern region of country, divided into two cohorts, patients and controls.

*Patients*. The ninety-three individuals all had a clinical diagnosis of pulmonary tuberculosis and were seen by the Pneumology Service of the Hospital José Ignacio Baldó,* Algodonal, Caracas*. There were 52 men (56%) and 41 women (44%), with an age range of 17–70 years. Patients were selected based on the diagnostic criteria of the National Standard Integrated Venezuela Tuberculosis Control Program: two sputum smears positive for acid fast bacilli by microscopy, clinical symptoms characteristic of TB, and a chest X ray consistent with active TB disease.

*Controls*. There were one hundred and two apparently healthy individuals who were known to be exposed to patients with active TB. This group was composed of staff members who had worked at Hospital Dr. José Ignacio Baldó, Algodonal, Caracas, for more than 3 years, and included 25 men (24.5%) and 77 women (75.5%) whose age range was 20–67 years. The controls were classified according to the tuberculin skin test “TST” as follows: positive TST (51/102), negative TST (19/102), or without information (32/102). All controls were without clinical manifestations of TB at the time of blood sampling.

Individuals, who were HIV positive or known to have any autoimmune, chronic inflammatory, or other disease, were excluded from the study. Participants in the study signed an informed consent form previously approved by the IVIC Bioethics Committee.

### 2.2. *NRAMP1* and* VDR* Genotype Analysis

Genomic DNA was extracted from blood samples according to the procedure described by Bunce [3]. The polymorphic variants of the* NRAMP1* gene were studied by PCR-RFLP technique, using primers and restriction enzymes reported by Taype et al., 2006 [4]:* INT4* (469 + 14G/C),* D543* (codon 543, Arg → Asp), and* 3*′*UTR* (deletion of TGTG in the 3′UTR, 55 nt 3′ to the last codon in exon 15). The polymorphic variants of the* VDR* gene were studied by PCR-RFLP, using primers and restriction enzymes reported by Curran et al., 1999 [5]: FokI, ApaI, and TaqI.

### 2.3. Statistical Analysis

Allele and genotype frequencies were determined by direct counting. The Hardy-Weinberg equilibrium was calculated with the exact test. The statistical significance of allele frequency differences between patients and controls was estimated by Fisher's exact test using 2 × 2 contingency tables. The Bonferroni corrected *p* values (*p*_c_) were obtained by multiplying the *p* values by the total number of variables analyzed and were considered significant when *p* < 0.05 [6]. Relative risk with corresponding 95% confidence intervals (95% CI) was calculated as odds ratios (OR) according to Woolf's formula [7].

## 3. Results

### 3.1. Frequency of* NRAMP1* Polymorphisms in Patients with Tuberculosis and Controls

Table 1 shows the frequencies of* NRAMP *genotypes and alleles in controls and TB patients. The allelic and genotypic frequencies of the* NRAMP1* polymorphisms showed Hardy-Weinberg equilibrium for* INT4* (*p* = 0.832),* D543* (*p* = 0.296), and* 3*′*UTR* (*p* = 0.594) polymorphisms in the control cohort. There was a significantly increased frequency of homozygous* 3*′*UTR* TGTG+/+ genotype in the healthy controls compared to the TB patients (79% versus 64%, resp., OR = 0.4, 95% CI: 0.2478–0.9045, *p* = 0.01, *p*_c_ = 0.03). However, the frequency of the heterozygous* 3*′*UTR* SNP TGTG+/del (32.6% versus 21%, resp., OR = 1.8, 95% CI: 0.9452–3.4976, *p* = 0.03) and homozygous* 3*′*UTR* SNP del/del genotypes (3.4% versus 0%, resp., OR = 8.13, 95% CI: 0.4143–159.6275, *p* = 0.02) was higher in the patient group than in the control group, although the corrected *p* values (*p*_c_) were not significant. In addition, there was a significant difference in the distribution of the allele frequencies (TGTG+ and TGTG del) between the controls and the TB patients (*p*_c_ = 0.018). There was no significant association between* INT4* and* D543* genotypes with tuberculosis.

### 3.2. Frequency of the* VDR* Polymorphism in Patients with Tuberculosis and Controls

Table 2 shows the frequencies of* VDR* genotypes and alleles in controls and TB patients. There was Hardy-Weinberg equilibrium for the genotype distributions of* FokI* (*p* = 0.074),* TaqI* (*p* = 0.066), and* ApaI* (*p* = 0.545) polymorphisms in apparently healthy individuals. The data showed that the frequency of the FF SNP genotype of* FokI* was higher in the patients than in the control group (36.6% versus 25.5%, resp., OR = 1.7, 95% CI: 0.9120–3.1109, *p* = 0.04) although the corrected *p* value was not significant. No significant difference in the allele frequencies was observed between the TB patients and the controls.

### 3.3. Genotype and Allele Distribution of* NRAMP-3*′*UTR* Variants in Patients with Tuberculosis and Healthy Controls Classified by the Tuberculin Skin Test (TST)

In order to investigate the possible influence of* NRAMP1-3*′*UTR* variants in the development of tuberculosis, we compared the genotype and allele frequencies between the different groups: TB patients versus TST positive controls, TB patients versus TST negative controls, and TST positive controls versus TST negative controls (Table 3). There were statistically significant differences between TB patients and TST negative controls for TGTG+/+ (64 versus 94.7%, OR: 0.1, 95% CI: 0.0126–0.7760, *p* = 0.004, *p*_c_ = 0.012) and TGTG+/del genotypes (32.6 versus 5.3%, OR: 8.7, 95% CI: 1.1069–68.3801, *p* = 0.008, *p*_c_ = 0.016). These same differences were conserved when the comparison was made between TST positive controls versus TST negative controls, although the significance was lost with the correction of *p* values. Additionally, there was a significant increase in the frequency of the TGTGdel allele among TB patients (OR: 9.0, 95% CI: 1.2010–68.2837, *p* = 0.005, *p*_c_ = 0.010) and TST positive controls (OR: 5.0, 95% CI: 0.6330–40.2130, *p* = 0.046, *p*_c_ = not significant) compared to TST negative controls.

## 4. Discussion

The aim of the present study was to look for associations between polymorphisms in* VDR* and* NRAMP1* genes and susceptibility to infection and disease with* Mycobacterium tuberculosis*, as indicated by a positive TST, and the development of TB in the Venezuelan population. Vitamin D is an immune-modulator molecule that, via its receptor VDR, can modulate cytokine responses by T cells [8]. Although several publications have reported an association between the* VDR* polymorphisms and tuberculosis in different populations [8–26], our study, similar to others [27–31], did not observe a significant association between the* ApaI*,* TaqI*, or* FokI* variants and susceptibility to either infection or development of tuberculosis.

Nramp1 (natural resistance-associated macrophage protein) is an integral membrane protein expressed exclusively in the lysosomal compartment of monocytes and macrophages. After phagocytosis, Nramp1 is targeted to the membrane of the microbe-containing phagosome, where it may modify the intraphagosomal milieu to affect microbial replication [32]. Some polymorphisms in the* NRAMP1* gene appear to favor bacterial replication within macrophages and have been associated not only with increased susceptibility to infection by* Mycobacterium tuberculosis* but also with an increased tendency to develop severe disease [33–35].

However, different studies have found conflicting results. There was a positive association between the* NRAMP1* gene* 3*′*UTR* polymorphism and susceptibility to tuberculosis in West Africans [36], Koreans [37], Chinese Han [23], and Chinese Kazak populations [38], but there was no association found in Taiwanese [39], Thai [40], Moroccan [41], Danish [42], Brazilian [43], and Indonesian [44] populations, and it was associated with resistance to TB in Cambodians [31]. The results presented here found that, in the Venezuelan population, the 3′UTR variants were associated with susceptibility to* M. tuberculosis* infection, as seen in the comparisons between TST+ and TST− controls, and also with progression to TB disease, as shown in the comparisons between TB patients and TST+ controls.

The* 3*′*UTR* polymorphism consists of a 4 bp TGTG deletion located 55 nucleotides downstream the last codon in exon 15 of the* NRAMP1* gene, in a region where sequence variation can affect mRNA stability and/or efficiency of protein translation. Therefore, to explain the increased susceptibility promoted by the TGTG+/del genotype and TGTGdel allele, two essential aspects should be considered: (1) the protein encoded by the* NRAMP1* gene plays an important role in the phagolysosomal function of pulmonary macrophages and in antigen presentation. The Nramp1 protein becomes activated and fused with lysosomes to digest the engulfed mycobacteria (reviewed in [45]); (2) Nramp1 pumps iron out of macrophages, thereby reducing iron levels within both the cytoplasm and the phagolysosome, rendering the metal less available for the iron-requiring intracellular bacilli [46]. As a consequence, a mutation or polymorphism in the* NRAMP1* gene that results in a nonfunctional Nramp1 protein or decreases production of the protein could cause a reduction in Nramp1 function or even a complete absence of the protein. Decreased Nramp1 action could lead to increased bacterial availability of iron, thereby promoting mycobacterial replication within macrophages. Because the iron is also required by the cell to generate reactive oxygen and nitrogen intermediates, the loss of Fe^2+^ ion transporter function of Nramp1 protein could increase availability of iron for intramacrophage bacteria and simultaneously weaken antimicrobial activity, thus favoring infection with* M. tuberculosis* and progression to tuberculosis disease. Furthermore, although elevated iron may increase susceptibility to TB, it may also predispose an individual to greater morbidity after TB has developed due to its role in generating ROS caused oxidative stress, which is greater in active TB compared with historical TB or healthy controls (reviewed in [46]). In conclusion the* NRAMP1* gene* 3*′*UTR* polymorphism might play an important role in the host defense to the development of tuberculosis.

## Figures and Tables

**Table 1:** Genotype and allele frequency distribution of *NRAMP1* gene in the controls and TB patients.

*INT4*	CC	GC	GG	Total
Genotypes				
*Controls* (number of individuals)	14	45	39	98
% of total	7.49	24.06	20.86	52.41
% within condition	14.29	45.92	39.80	
% within *INT4*	60.87	56.25	46.43	

*TB patients* (number of individuals)	9	35	45	89
% of total	4.81	18.72	24.06	47.59
% within condition	10.11	39.33	50.56	
% within *INT4*	39.13	43.75	53.75	

*Total*	23	80	84	187
% of total	12.30	42.78	44.92	100

*χ* ^2^ (df: 2) = 2.34 *p* = 0.3107 Cramer's *V* = 0.1118				

*D543*	AA	AG	GG	Total
Genotypes				

*Controls* (number of individuals)	1	10	89	100
% of total	0.52	5.21	46.35	52.08
% within condition	1	10	89	
% within *D543*	25	55.56	52.35	

*TB patients* (number of individuals)	3	8	81	92
% of total	1.56	4.17	42.19	47.92
% within condition	3.3	8.7	88	
% within *D543*	75	44.44	47.65	

*Total*	4	18	170	192
% of total	2.08	9.38	88.54	100

*χ* ^2^ (df: 2) = 1.27 *p* = 0.529 Cramer's *V* = 0.0813				

*3'UTR*	TGTGdel/del	TGTG+/del	TGTG+/+	Total
Genotypes				

*Controls* (number of individuals)	0	21	79	100
% of total	0.00	11.11	41.80	52.91
% within condition	0.00	21.00	79.00	
% within *3'UTR*	0.00	42.00	58.09	

*TB patients* (number of individuals)	3	29	57	89
% of total	1.59	15.34	30.16	47.09
% within condition	3.37	32.58	64.04	
% within *3'UTR*	100.00	58.00	41.91	

*Total*	3	50	136	189
% of total	71.96	26.46	1.59	100

*χ* ^2^ (df: 2) = 7.22 *p* = 0.0270 Cramer's *V* = 0.1955				

*Note*. *p*: probability values; + = presence of TGTG; del = absence of these four bases; df: degree freedom; Cramer's *V*: measure of association between two variables.

**Table 2:** Genotype and allele frequency distribution of *VDR g*ene in the controls and TB patients.

*Fok1*	ff	Ff	FF	Total
Genotypes				
*Controls* (number of individuals)	16	60	26	102
% of total	8.2	30.8	13.3	52.3
% within condition	15.7	58.8	25.5	
% within *Fok1*	57.1	56.1	43.3	

*TB patients* (number of individuals)	12	47	34	93
% of total	6.2	24.1	17.4	47.7
% within condition	12.9	50.5	36.6	
% within *Fok1*	42.9	43.9	56.7	

*Total*	28	107	60	195
% of total	14.4	54.9	30.7	100

*χ* ^2^ (df: 2) = 2.81 *p* = 0.2454 Cramer's *V* = 0.12				

*Taq1*	tt	Tt	TT	Total
Genotypes				

*Controls* (number of individuals)	1	38	58	97
% of total	0.5	20.8	31.7	53
% within condition	1	39.2	59.8	
% within *Taq1*	33.3	53.5	53.2	

*TB patients* (number of individuals)	2	33	51	86
% of total	1.1	18	27.9	47
% within condition	2.3	38.4	59.3	
% within *Taq1*	66.7	46.5	46.8	

*Total*	3	71	109	183
% of total	1.6	38.8	59.6	100

*χ* ^2^ (df: 2) = 0.48 *p* = 0.7866 Cramer's *V* = 0.0512				

*Apa1*	aa	Aa	AA	Total
Genotypes				

*Controls* (number of individuals)	18	54	29	101
% of total	9.5	28.4	15.3	53.2
% within condition	17.8	53.5	28.7	
% within *Apa1*	47.4	56.3	51.8	

*TB patients* (number of individuals)	20	42	27	89
% of total	10.5	22.1	14.2	46.8
% within condition	22.5	47.2	30.3	
% within *Apa1*	66.7	46.5	46.8	

*Total*	38	96	56	190
% of total	20	50.5	29.5	100

*χ* ^2^ (df: 2) = 0.92 *p* = 0.6313 Cramer's *V* = 0.0696				

*Note*. *p*: probability values; df: degree freedom; Cramer's *V*: measure of association between two variables.

**Table 3:** Genotype and allele frequency distribution of *NRAMP-3*′*UTR *in TB patients and healthy controls grouped according to the TST.

*3*′*UTR*	TGTGdel/del	TGTG+/del	TGTG+/+	Total
Genotypes				
*Controls TST*+ (number of individuals)	0	12	38	50
% of total	0.00	8.6	27.3	36
% within condition	0.00	24.00	76.00	
% within *3'UTR*	0.00	29.3	40	

*TB patients* (number of individuals)	3	29	57	89
% of total	2.2	20.9	41	64
% within condition	3.4	32.6	64	
% within *3'UTR*	100.00	70.7	60	

*Total*	3	41	95	139
% of total	2.2	29.5	68.3	100

*χ* ^2^ (df: 2) = 3.15 *p* = 0.207 Cramer's *V* = 0.1505				

*3*′*UTR*	TGTGdel/del	TGTG+/del	TGTG+/+	Total
Genotypes				

*Controls TST*− (number of individuals)	0	1	18	19
% of total	0.00	0.9	16.7	17.6
% within condition	0.00	5.3	94.7	
% within *3'UTR*	0.00	3.3	24	

*TB patients* (number of individuals)	3	29	57	89
% of total	2.8	26.9	52.8	82.4
% within condition	3.4	32.6	64	
% within *3'UTR*	100.00	96.7	76	

*Total*	3	30	75	108
% of total	2.8	27.8	69.4	100

*χ* ^2^ (df: 2) = 6.97 *p* = 0.0307 Cramer's *V* = 0.254				

*3*′*UTR*	TGTGdel/del	TGTG+/del	TGTG+/+	Total
Genotypes				

*Controls TST*− (number of individuals)	0	1	18	19
% of total	0.00	1.4	26.1	27.5
% within condition	0.00	5.3	94.7	
% within *3'UTR*	0.00	7.7	32.1	

*Controls TST*+ (number of individuals)	0	12	38	50
% of total	0.00	17.4	55.1	72.5
% within condition	0.00	24.00	76.00	
% within *3'UTR*	0.00	92.3	67.9	

*Total*	0	13	56	69
% of total	0	18.8	81.2	100

*χ* ^2^ (df: 2) = 3.16 *p* = 0.206 Cramer's *V* = 0.214				

*Note*. *p*: probability values; df: degree freedom; Cramer's *V*: measure of association between two variables.
